# Comparing the outcomes of robotic assisted radical prostatectomy in black and white men: Experience of a high-volume center

**DOI:** 10.1590/S1677-5538.IBJU.2022.9979

**Published:** 2022-10-03

**Authors:** Jonathan Noël, Marcio Covas Moschovas, Marco Sandri, Abdel Rahman Jaber, Travis Rogers, Vipul Patel

**Affiliations:** 1 AdventHealth Global Robotics Institute Celebration FL USA AdventHealth Global Robotics Institute, Celebration, FL, USA; 2 Guy's and St Thomas’ NHS Foundation Trust London UK Guy's and St Thomas’ NHS Foundation Trust, London, UK; 3 University of Central Florida Orlando FL USA University of Central Florida (UCF), Orlando, FL, USA; 4 University of Brescia Big and Open Data, Innovation Laboratory Brescia Italy Big and Open Data, Innovation Laboratory, University of Brescia, Brescia, Italy

**Keywords:** Robotic Surgical Procedures, Prostatectomy, Prostatic Neoplasms

## Abstract

**Background:**

Global cancer incidence ranks Prostate Cancer (CaP) as the second highest overall, with Africa and the Caribbean having the highest mortality. Previous literature suggests disparities in CaP outcomes according to ethnicity, specifically functional and oncological are suboptimal in black men. However, recent data shows black men achieve post radical prostatectomy (RP) outcomes equivalent to white men in a universally insured system. Our objective is to compare outcomes of patients who self-identified their ethnicity as black or white undergoing RP at our institution.

**Materials and methods:**

From 2008 to 2017, 396 black and 4929 white patients underwent primary robotic-assisted radical prostatectomy (RARP) with a minimum follow-up of 5 years. Exclusion criteria were concomitant surgery and cancer status not available. A propensity score (PS) match was performed with a 1:1, 1:2, and 1:3 ratio without replacement. Primary endpoints were potency, continence recovery, biochemical recurrence (BCR), positive surgical margins (PSM), and post-operative complications.

**Results:**

After PS 1:1 matching, 341 black vs. 341 white men with a median follow-up of approximately 8 years were analyzed. The overall potency and continence recovery at 12 months was 52% vs 58% (p=0.3) and 82% vs 89% (p=0.3), respectively. PSM rates was 13.4 % vs 14.4% (p = 0.75). Biochemical recurrence and persistence PSA was 13.8% vs 14.1% and 4.4% vs 3.2% respectively (p=0.75). Clavien-Dindo complications (p=0.4) and 30-day readmission rates (p=0.5) were similar.

**Conclusion:**

In our study, comparing two ethnic groups with similar preoperative characteristics and full access to screening and treatment showed compatible RARP results. We could not demonstrate outcomes superiority in one group over the other. However, this data adds to the growing body of evidence that the racial disparity gap in prostate cancer outcomes can be narrowed if patients have appropriate access to prostate cancer management. It also could be used in counseling surgeons and patients on the surgical intervention and prognosis of prostate cancer in patients with full access to gold-standard screening and treatment.

## INTRODUCTION

Global cancer statistics (GLOBOCAN 2020) (1) have ranked prostate cancer (CaP) as the third highest organ site for new cases and the third highest cause of death due to cancer among both genders. In men specifically, CaP is second to lung cancer in incidence, with Africa and the Caribbean having the highest mortality due to the former. The literature has suggested that racial disparity in CaP outcomes exists owing to differences in socioeconomics, access to healthcare (2); genetic profiles (3), pathological upstaging after radical prostatectomy (RP) (4), and surgical acumen (5). Previous studies have reported that the functional and oncological outcomes are suboptimal in black men, and as a result, their CaP management requires a refined approach (6).

Parry et al. (7) confirmed in a UK National CaP Audit that the likelihood of receiving radical treatment was higher in more affluent, lower comorbid, and non-black patients. The comparative outcomes per ethnic category are awaited as the evidence of comparable response to standard of care CaP treatment increases. Cole et al. (8) analyzed data from Massachusetts, US, to investigate comparative time to definitive treatment within 90 days of diagnosis (RP or radiation therapy [RT]) and cancer-specific survival. The study found that black men received less definitive treatment and had lower cancer-specific mortality than white men, concluding that both ethnic groups achieved equivalent outcomes in universally insured health locations. This is supported by a meta-analysis of seven randomized clinical trials (9), where the response of black men to radiotherapy (RT) was superior to white men. Considering the above, CaP management should result in equivalent or even superior outcomes in black men compared to matched groups in some circumstances.

The increased use of robotic approaches has favorably impacted the urinary and sexual function profile of patients compared to open and laparoscopic surgery (10). Additionally, the intra and postoperative complication rates are less with RARP than with open RP (11). Therefore, we aim to compare the outcomes of patients with full access to healthcare who self-identified their ethnicity as black or white undergoing RARP in our institution.

### Materials and Methods

#### Study population

We used our Institutional Review Board-approved (number 237998) prospectively maintained database. We included 396 self-identified black patients from 2008 to 2017 and compared to 4929 self-identified white patients operated on during the same period. We considered patients who underwent primary RARP with a minimum follow-up of 5 years. We excluded patients who underwent dialysis, kidney transplant recipients, and salvage RARP after focal therapy or RT, concomitant abdominal pelvic surgery such as hernia repair or appendectomy (black n =26, white n = 340), and cancer status unknown or not available (black n= 29, white n =249).

#### Propensity Score Matching

Controlling for baseline differences, 341 black patients were propensity score (PS) matched in a 1:1, 1:2. 1:3 ratio from a cohort of 4340 white patients. Using a multiple variable logistic regression model for PS, based on: age, prostate specific antigen (PSA) levels, body mass index (BMI), Charlson comorbidity index (CCI), CCI in 3 groups, Sexual Health Inventory for Men (SHIM) score, American Urological Association symptom score (AUASS), AUASS in 3 groups, prostate specimen weight, PSA density, follow up time, year of RARP, biopsy International Society of Urological Pathology (ISUP) grade group, biopsy primary pattern Gleason score (GS), biopsy secondary pattern GS, biopsy total GS, clinical tumor stage, D’Amico risk classification, smoking history, family history of prostate cancer and breast cancer respectively.

Matching was performed using the nearest-neighbor matching algorithm (caliper width 0.15 of the standard deviation of the logit score) with a 1:1 ratio without replacement (12). The balance diagnostics applied to covariates was standardized mean differences comparing before and after PS matching in the two groups (13).

### Statistical Analysis

Using established guidelines (14) continuous variables were reported as median and interquartile range (IQR) and categorical variables as absolute and relative frequencies. A two-sample Wilcoxon rank-sum test was used for testing the hypothesis of equal distributions in the matched groups for continuous variables. The Fisher's exact test compared the groups for categorical variables. For continuous outcomes, the confidence intervals (CIs) for the difference between the two study groups median was performed with Hodges-Lehmann method (15). Cumulative incidence functions (CIF) for post-operative recovery of potency, continence, and biochemical recurrence (BCR) were estimated by the Kaplan-Meier method.

The STSURVDIFF module (16) in Stata was used to estimate the difference of cumulative incidences groups (with CIs) between study groups for time-to-event outcomes at fixed time points after RARP. The statistical analyses were performed using Stata 16 and R version 4.0.2 (Stata Corp., College Station, TX, US). Statistical significance is defined as p < 0.05 for a two-tailed test.

#### Surgical technique

All patients underwent RARP by a single surgeon via a six-port configuration, transperitoneal retropubic approach, using the da Vinci surgical system (Intuitive Surgical, Sunnyvale, CA, USA) with our previously described techniques (17–20). We used athermal retrograde release of neurovascular bundles and posterior reconstruction (12).

#### Endpoints

Primary endpoints were the comparative Pentafecta for the attainment of continence and potency, BCR, postoperative early complications, and positive surgical margins (PSM) between the groups. Continence is defined as the use of no pads, potency as achieving and maintaining erections sufficient to perform intercourse (with or without phosphodiesterase type 5 inhibitor use), and BCR is defined as a postoperative PSA above 0.2 ng/mL. Additionally, we compared the peri-operative characteristics of the groups.

Secondary endpoints of the study were comparing groups of the time to hormonal, radiotherapy, and chemotherapy, where applicable.

## RESULTS

Table-1 demonstrates part of the variables before (341 vs. 4340) and after PS 1:1 matching (341 vs. 341). Median follow-up was 2915 vs. 2917 days or approximately 8 years in both groups. Perioperative findings are presented in Table-2, with similar estimated blood loss (EBL) and median console time for both groups. The median operative time was 5 minutes longer in the black patient group (p=0.02). The maximum hospital stay was 13 days for black patients and 6 days for white patients, with the median time being 1 day in both groups. The complication rates classified by the Clavien-Dindo scale, and 30-day readmission rate did not differ among the groups.

**Table 1:** Comparison of variables for study groups prior to and after 1:1 PS match. IQR (Interquartile range), PS (propensity score), SDD (standardized difference), PSA (prostate specific antigen), BMI (body mass index), CCI (Charlson comorbidity index), SHIM (Sexual Health Inventory for Men), AUASS (American Urological Association symptom score), ISUP (International Society of Urological Pathology).

Variable		Black Patients (N=341)	Before PS matching			After 1:1 PS matching		
			White Patients (n=4340)	P value	SDD	White Patients (n=341)	P value	SDD
Age, years (IQR)		59 (53-63)	62 (56-67)	<0.001	0.41	59 (52-64)	0.99	-0.01
PSA, ng/mL (IQR)		5.3 (4.3-7.8)	5.1 (4-6.9)	0.113	-0.96	5.4 (4.3-7.6)	0.89	0.07
BMI, kg/m^2^(IQR)		28.4 (25.9-31.6)	27.6 (254-30.4)	0.005	-0.22	27.8 (25.5-31.2)	0.12	-0.13
CCI score (IQR)		2 (1-2)	2 (1-2)	0.003	0.24	2 (1-2)	1	0.03
**CCI, n (%)**								
	0-1	152 (44.6)	1499 (34.5)	<0.001	-2.1	150 (43.9)	0.5	-0.01
	2-3	180 (52.8)	2615 (60.3)		0.15	176 (51.6)		-0.02
	**≥4**	9 (2.6)	226 (5.2)		1.33	15 (4.4)		0.09
SHIM score (IQR)		20 (15-24)	21 (15-25)	0.24	-0.028	22 (16-25)	0.24	0
Prostate weight grams (IQR)		51 (42-62)	48 (40-60)	0.025	-0.12	49 (41-60)	0.54	-0.12
PSA Density		0.10 (0.08-0.15)	0.10 (0.07-0.14)	0.37	-0.05	0.12 (0.08-0.16)	0.14	0.12
AUASS (IQR)		6 (2-11)	7 (3-12)	0.14	0.07	6 (3-11)	0.98	-0.02
Follow up, days (IQR)		2915 (2193-3646)	2925 (2222-3647)	0.27	0.11	2917(2249-3309)	0.32	0.03
**Biopsy ISUP grade group, n (%)**								
	Grade group 1	157 (46)	2018 (46.5)	0.6	0.01	166 (48.7)	0.9	0.05
	Grade group 2	112 (32.8)	1306 (30.1)		-0.06	110 (32.7)		-0.01
	Grade group 3	30 (8.8)	495 (11.4)		0.09	26 (7.6)		-0.04
	Grade group 4	28 (8.2)	344 (7.9)		-0.01	28 (8.2)		0
	Grade group 5	14 (4.1)	177 (4.1)		-0.001	11 (3.2)		-0.05
**Clinical stage, n (%)**								
	T1a	1 (0.3)	7 (0.2)	0.05	-0.03	0 (0)	0.9	-0.08
	T1b	0 (0)	2 (0.1)		0.03	0 (0)		
	T1c	284 (83.3)	3358 (77.4)		-0.15	275 (80.7)		-0.07
	T2a	41 (12)	706 (16.3)		0.12	51 (14.9)		0.09
	T2b	5 (1.5)	150 (3.5)		0.13	5 (1.5)		0
	T2c	8 (2.4)	89 (2.1)		-0.02	8 (2.4)		0
	T3a	1 (0.3)	25 (0.6)		0.04	1 (0.3)		0
	T3b	0 (0)	2 (0.1)		0.03	0 (0)		0
	T4	1 (0.3)	1 (0.02)		-0.07	1 (0.3)		
**D’Amico Risk, n (%)**								
	Low	144 (42.2)	1820 (41.9)	0.3	0.01	145 (42.5)	0.75	0.01
	Intermediate	137 (40.2)	1871 (43.1)		0.06	143 (41.9)		0.04
	High	60 (17.6)	649 (15)		-0.07	53 (15.5)		-0.06
**Smoking, n (%)**								
	No	273 (80.1)	3189 (73.5)	0.001	-0.15	279 (81.8)	0.78	0.05
	Yes, former	42 (12.3)	872 (20.1)		0.212	36 (10.6)		-0.06
	Yes, current	26 (7.6)	279 (6.4)		-0.05	26 (7.6)		0
**Family History CaP, n (%)**								
	No	202 (59.2)	3793 (87.4)	0.03	0.12	203 (59.5)	1	0.01
	Yes	139 (40.8)	547 (12.6)		-0.12	138 (40.5)		-0.01
**Family History Breast Ca, n (%)**								
	No	308 (90.3)	3793 (87.4)	0.12	-0.93	290 (85)	0.05	-0.16
	Yes	33 (9.7)	547 (12.6)		0.09	51 (15)		0.16

**Table 2:** Peri-operative outcomes for study groups, 1:1 PS match. EBL (estimated blood loss), IQR (Interquartile range), PS (propensity score).

Perioperative		Black Patients (N=341)	After 1:1 PS matching	
			White Patients (n=341)	P value
EBL mL, (IQR)		100 (75-150)	100 (100-150)	0.83
Operative time, minutes (IQR)		123 (110-138)	119 (106-133)	0.02
Console time, minutes (IQR)		75 (75-80)	75 (75-80)	0.15
In-hospital stay, days, n (IQR)		1 (1-1)	1 (1-1)	0.06
**Clavien Dindo, n (%)**				
	0	314 (92)	323 (94.7)	0.4
	1	9 (2.6)	8 (2.4)	
	2	15 (4.4)	7 (2.1)	
	3	1 (0.3)	2 (0.6)	
	4	2 (0.6)	1 (0.3)	
Readmission <30 days, n (%)		5 (1.47)	4 (1.17)	0.5

Pathological and cancer status outcomes are described in Table-3. PSM and ECE events were 13.4 % vs 14.4% (p = 0.75) and 23.8% vs 27.3% (p=0.3) respectively. BCR and persistent PSA (or PSA that did not decrease to <0.1ng/mL post RARP) was 13.8% vs 14.1% and 4.4% vs 3.2% respectively (p=0.75).

**Table 3:** Pathological and Oncological outcomes for study groups in 1:1 PS match. IQR (Interquartile range), PS (propensity score), PSM (positive surgical margin), ECE (extracapsular extension), BCR (biochemical recurrence), ISUP (International Society of Urological Pathology).

Cancer outcome		Black Patients (N=341)	After 1:1 PS matching	
			White Patients (n=341)	P value
**ISUP grade group, n (%)**				
	1	88 (27.6)	100 (29.3)	0.4
	2	160 (46.9)	160 (46.9)	
	3	65 (19.1)	53 (15.5)	
	4	8 (2.4)	13 (3.8)	
	5	20 (5.9)	15 (4.4)	
**Tumor upgrade, n (%)**				
	Yes	145 (42.5)	148 (43.4)	0.8
	No	196 (57.5)	193 (56.6)	
PSM, mm (IQR)		2 (1-3)	2 (1-3)	0.9
PSM present, n (%)		46 (13.5)	49 (14.4)	0.8
ECE, mm (IQR)		1 (1-2)	1 (1-2)	0.9
ECE present, n (%)		81 (23.8)	93 (27.3)	0.3
Tumor volume % (IQR)		15 (10-20)	15 (8-20)	0.4
**Pathological Stage, n (%)**				
	≤T2c	50 (14.6)	64 (18.7)	0.4
	T3a	2 (0.6)	1 (0.39)	
	T3b	229 (67.2)	210 (61.6)	
	T4	60 (17.6)	66 (19.4)	
Positive lymph node, n (IQR)		0 (0-0)	0 (0-0)	0.6
**Cancer Status, n (%)**				
	BCR	47 (13.8)	48 (14.1)	0.75
	Persistent PSA	15 (4.4)	11 (3.2)	

**Figure 1:**
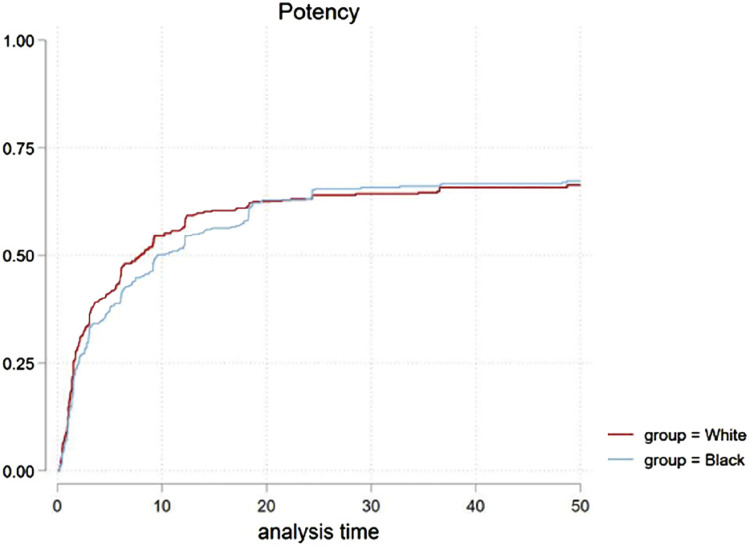
CIF of potency recovery for both groups in 1:1 PS match, P=0.3.

Figure-1 describes the cumulative incidence function (CIF) for potency recovery between groups (p=0.3). A Cox regression sub-analysis for potency recovery showed a statistically significant difference in white patients if pre-RARP SHIM was >17 (p=0.04), but not if SHIM was ≤17 (p=0.16). Patients with pre-operative SHIM >22 (p=0.4), SHIM ≤22 (p=0.9), age at RARP >65 (p=0.7) or ≤65 (p=0.1) did not have statistically significant differences in potency recovery.

**Figure 2:**
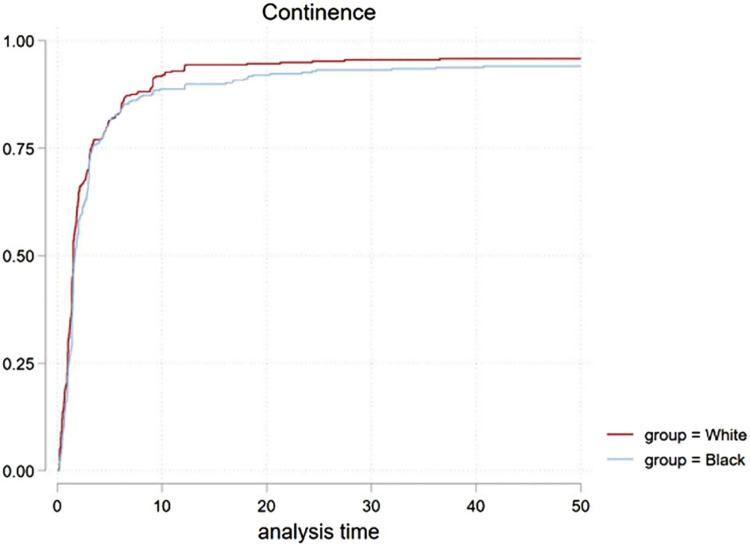
CIF of continence recovery for both groups in 1:1 PS match, P=0.3.

**Figure 3:**
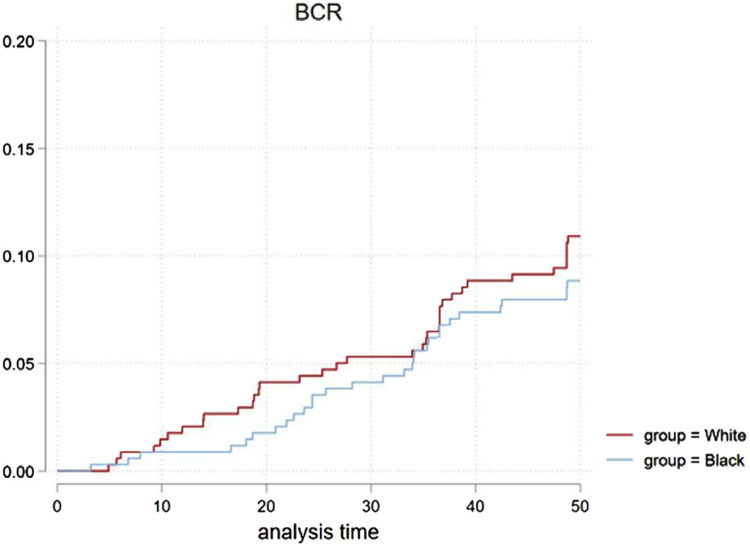
CIF for BCR for both groups in 1:1 PS match, P=0.9.

The continence recovery CIF curve (Figure-2) showed a non-significant statistical difference in recovery (p=0.3) overall. Further comparative analysis performed based on age, with patients >65 (p=0.25) and ≤65 years old (p= 0.051) was similar between groups. BCR rates over time was compared in Figure-3 (p=0.9). Only one death for cancer was observed (white patient group), hence cancer specific survival cannot be analyzed in our study.

The overall potency and continence recovery at 12 months was 52% vs 58% (p=0.3) and 82% vs 89% (p=0.3) respectively. In Table-4, the potency and continence recovery, along with BCR, are demonstrated at set time points with cumulative rates for each group. The groups’ cumulative rate differences with associated CI for each time point are shown.

**Table 4:** Time to event Functional and BCR outcomes for study groups in 1:1 PS match. CI = confidence interval, HR=hazard ratio versus black patients as reference. s

Outcome		Cumulative rate (%)		Difference, % (95% CI) *	HR (95% CI)	P value**
		Black Patients (N=341)	White Patients (n=341)			
**Potency, months**						
	3 months	31	39	7.7 (0.6 – 14.9)	0.9 (0.7 – 1.1)	0.3
	6 months	39	47	7.2 (-3 – 14.6)		
	9 months	46	54	8.4 (0.9 – 15.9)		
	12 months	52	58	6.3 (-1.1 – 13.8)		
	24 months	63	67	3.4 (3.7 – 10.6)		
	36 months	66	68	1.4 (-5.7 – 8.4)		
**Continence**						
	3 months	68	69	1.8 (-5.1 – 8.6)	0.9 (08 – 1.1)	0.3
	6 months	83	84	-0.3 (-5.8 – 5.2)		
	9 months	88	90	2.9 (-1.7 – 6.6)		
	12 months	89	92	2.3 (-1.9 - 6.6)		
	24 months	93	94	1.2(-2.5 – 4.9)		
	36 months	94	94	0.6 (-2.9 – 4.1)		
**BCR**						
	3 months	0	0	-0.3 (-0.9 – 0.3)	0.9 (0.7 – 1.5)	0.9
	6 months	0.3	0.6	0 (11.1 – 1.1)		
	9 months	0.9	0.9	0.3 (-1.2 – 1.8)		
	12 months	0.9	1	0.9 (-0.8 – 2.6)		
	24 months	3	4	0 (-2.8 – 2.8)		
	36 months	6	5	-2.1 (-5.5 – 1.4)		

* Difference as white – black

** Long-rank test to compare cumulative incidence functions.

## DISCUSSION

Literature has cited black ethnicity as a prognostic factor for adverse pathological features and higher PSM rates compared to matched white patients (21, 22). However, the wider utilization of PSA, patient awareness, and acceptance of screening for CaP contributed to a stage shift. The BCR-free survival gap has narrowed between these two ethnic groups in the US (23). Riviere et al. (24) showed from a VA database of 20 million veterans that 60,000 black and white men with equal access to care experienced similar outcomes. However, on the other side, a literature review (25) of men of African descent found CaP data from the Caribbean and the UK differed from the US. The UK's initiative of the National CaP Audit can now assess the impact of universal access from the National Health Service (NHS) on the outcomes of RP according to ethnicity. Furthermore, in some countries, COVID-19 negatively impacted on increasing advanced CaP presentations and a reduction in RP and RT by 26.9% and 14.1%, respectively (26).

Our study investigated the results of a high-volume center and a single surgeon with expertise in RARP among patients who self-identified as black and white ethnicity. Although our institute receives referrals of patients post-Focal and Radiation therapy for Salvage RARP, they were excluded due to significantly variable and generally inferior outcomes compared to primary surgery (27). Further exclusions were abdominal and pelvic surgery due to the potential impact on perioperative and nerve-sparing results (28). Given the high number of white patients in our databank, we were able to consider numerous variables on the PS to improve the precision (29) and balance between the groups (30) before the analysis. It is known that family history and the association with the BRCA2 gene increase the risk of CaP (31). However, we could not consider these variables in the PS due to the reduced number of patients.

Regarding our comparative functional outcomes, we observed in Table-4 that the cumulative risk showed a rapid potency recovery in the first year after RARP, although not statistically significant between the groups. DeCastro et al. (32) analyzed their single center results in the first-year post RARP between black and non-black patients and found equivalency in both potency and continence at 6 months but inferior outcomes in black patients at 12 months. Another study attributed anatomical differences, such as median urethral length in Asian compared to non-Asian patients (33), to differences in Expanded Prostate Cancer Index Composite for Clinical Practice (EPIC-CP) urinary scores post-RP. Furthermore, Von Bodman et al. (34) compared MRI scans among ethnic groups to reveal a steeper symphysis pubis angle and mid-pelvic area in black patients. Despite these findings in previous studies, we found surgical outcomes equivalence between groups, including PSM rates, due to our standard surgical technique respecting anatomical landmarks. Meticulous surgical care must involve technique adaptability according to the tumor burden and biopsy histology (35).

Our patients with biopsy ISUP 1, who elected RP overactive surveillance, made up the majority of the cohort. A previous study from John Hopkins (36) found that black men with very low-risk CaP who underwent RP had disease upgrades and higher rates of PSM compared to other ethnic groups. This study suggested counseling black patients on their oncologic risks around treatment options. Individual cancer referral centers’ results vary based on practice parameters such as volume and individual surgeon experience. Our study could not demonstrate a clinically or statistically difference in tumor upgrades, PSM, and BCR between groups, suggesting that the oncologic outcomes are comparable in our expertise. BCR as a stand-alone (Figure-3) or with persistent PSA (Table-3) presented a statistically non-significant difference. This is also reported by a multicentric study (37) assessing ISUP 4 and 5 CaP, which concluded no difference in the rate of adverse oncologic outcomes by ethnicity.

The overall survival and cancer-specific survival were not analyzed due to the low event rate in our study. Data from the American College of Surgeons National Surgical Quality Improvement Program included 38,642 patients (38) undergoing major urological cancer surgery (RP, radical/partial nephrectomy [PN/RN], and radical cystectomy [RC]). It analyzed trends based on ethnicity and found no increase in the risk of 30-day postoperative complications between groups. After controlling for comorbidities, black ethnicity did not show independent association for complications in RP (odds ratio [OR] = 1.08, 95% CI: 0.92-1.29). Our results were also compatible with these findings and could not find differences between the groups in EBL, Clavien-Dindo complications, and readmission rates.

The oncological outcomes in a universal healthcare institution in the US have been previously found to be superior or similar as it eliminated access to healthcare barriers. Our referral center accepts privately insured, universally insured Medicare, US Veterans Affairs (VA) populations, and international patients. In our experience, we believe that this full healthcare access is crucial to minimize the difference in outcomes, similar to findings in a universal healthcare (8, 20), or single payer systems (10) due to appropriate prostate cancer screening with PSA, access to imaging exams such as Multiparametric Resonance Image (MRI), and follow-up according to established guidelines.

Despite its strengths, this study is limited by its retrospective design and all inherent risks of bias. Additionally, an absence of analysis from other ethnic cohorts and the unmeasured variation in self-identifying as white and black. Furthermore, this series reflects optimal outcomes of a high-volume expert single surgeon, and these results may not be reproducible in low-volume centers. However, to our knowledge, this is one of the largest cohorts in the literature comparing outcomes in patients from two ethnic groups with similar peri-operative characteristics (balanced with PS) and full access to gold-standard treatments for prostate cancer.

## CONCLUSIONS

In our study, comparing two ethnic groups with similar preoperative characteristics and full access to screening and treatment showed compatible RARP results. We could not demonstrate outcomes superiority in one group over the other. However, this data adds to the growing body of evidence that the racial disparity gap in prostate cancer outcomes can be narrowed if patients have appropriate access to prostate cancer management. It also could be used in counseling surgeons and patients on the surgical intervention and prognosis of prostate cancer in patients with full access to gold-standard screening and treatment.
